# Emerging role of glutathione peroxidase 4 in myeloid cell lineage development and acute myeloid leukemia

**DOI:** 10.1186/s11658-024-00613-6

**Published:** 2024-07-08

**Authors:** Patrick Auberger, Cécile Favreau, Coline Savy, Arnaud Jacquel, Guillaume Robert

**Affiliations:** 1 University of Nice Cote d’Azur (UniCA), Nice, France; 2 Mediterranean Centre for Molecular Medicine, C3M, Inserm U1065, Team 2 “Innovative Therapies in Myeloid Leukemia”, Nice, France; 3 Centre Hospitalier Universitaire, Nice, France

**Keywords:** GPX4, Ferroptosis, Myeloid differentiation, Myeloid leukemia, AML, Small molecule inhibitors, Therapeutic strategies

## Abstract

Phospholipid Hydroperoxide Gluthatione Peroxidase also called Glutathione Peroxidase 4 is one of the 25 described human selenoproteins. It plays an essential role in eliminating toxic lipid hydroxy peroxides, thus inhibiting ferroptosis and favoring cell survival. GPX4 is differentially expressed according to myeloid differentiation stage, exhibiting lower expression in hematopoietic stem cells and polymorphonuclear leucocytes, while harboring higher level of expression in common myeloid progenitors and monocytes. In addition, GPX4 is highly expressed in most of acute myeloid leukemia (AML) subtypes compared to normal hematopoietic stem cells. High GPX4 expression is consistently correlated to poor prognosis in patients suffering AML. However, the role of GPX4 in the development of the myeloid lineage and in the initiation and progression of myeloid leukemia remains poorly explored. Given its essential role in the detoxification of lipid hydroperoxides, and its overexpression in most of myeloid malignancies, GPX4 inhibition has emerged as a promising therapeutic strategy to specifically trigger ferroptosis and eradicate myeloid leukemia cells. In this review, we describe the most recent advances concerning the role of GPX4 and, more generally ferroptosis in the myeloid lineage and in the emergence of AML. We also discuss the therapeutic interest and limitations of GPX4 inhibition alone or in combination with other drugs as innovative therapies to treat AML patients.

## Background

Glutathione peroxidase 4 (GPX4) is a member of the GPX protein family that also comprised GPX 1, 2, 3, 5, 6, 7 and 8 in mammals. GPX1, 2, 3, 4 and 6 carry a highly reactive selenocysteine residue in their active site that is crucial for enzymatic activity and the efficient reduction of membrane hydroxy peroxides into their alcohol counterparts. GPX4 is a 20-30 kDa molecular weight protein depending on the nature of the expressed isoform. It behaves as a monomer composed of 4 α-helices and 7 β-strands. The conserved catalytic site comprises a tetrad of amino acids: Sec46, Gln81, Trp136 and Asn147, the latter being directly involved in catalysis. Replacement of Sec46 by a cysteine or mutation of any of the 3 other amino acids of the catalytic site results in an abrogation or a diminution of GPX4 enzymatic activity [1].

GPX4 is expressed in different cell types and tissues as three distinct cellular isoforms, namely mitochondrial GPX4 (mGPX4), cytoplasmic GPX4 (cGPX4), and nuclear GPX4 (nGPX4,) that are encoded by the same gene and generated using alternate promoters or alternative splicing. GPX4 is the only GPX indispensable for embryonic development and cell survival. Indeed, deletion of GPX1, GPX2, or GPX3 in mice fails to affect development and survival [2]. Of note, GPX4 deficient mice are not viable and die at embryonic day 8 [3, 4]. Moreover, only cGPX4 has been identified as essential for embryonic development and cell survival [1, 5]. Why only ablation of cGPX4 is lethal in mice remains an enigma. It may, however, be linked to the unique function of cGPX4 in the regulation of lipid peroxidation. Of note, conditional inducible deletion of GPX4 in neurons results in neurodegeneration consecutive to rapid cell death [6].

Various transcription factors are involved in GPX4 transcriptional regulation. They include CAMP-response element binding protein (CREB), SP1 transcription factor (SP1), transcription factor AP-2 (AP2), proliferator-activated receptor alpha (PPARα), all activating GPX4 expression, while Krüppel-like transcription factor 2 (KLF2) and CAMP-response element modulator (CREM), both repress its protein expression [7].

GPX4 can be also modified post-transcriptionally by different processes such as ubiquitination of several lysine residues including Lys 47, 80, 107, 135, 162, 167 and 191, phosphorylation of Ser13, 40 and Tyr96, but also succinylation at Cys93 or alkylation at Cys107. The precise role of each of these modifications for protein function remains unclear. Nevertheless, degradation appears to be the most common mechanism for GPX4 inactivation since many small molecule inhibitors can trigger it. Degradation can occur through several pathways, including the ubiquitin proteasome system (UPS) or various forms of autophagy, including macro-autophagy or chaperone-mediated autophagy (CMA). The role of different modes of autophagy in the degradation of GPX4 will be discussed latter in this review.

Among all GPXs proteins, GPX4 has gained lot of attention these past last years for its essential role in inhibiting ferroptosis, a non-apoptotic and iron-dependent mode of regulated cell death [8, 9]. GPX4 converts lipid hydroxy peroxides to corresponding alcohols or free hydrogen peroxide to water together with the reduction of glutathione (GSH) to oxidized glutathione (GSSG) and as such plays an essential role in protecting cells from oxidative stress and ferroptosis [10–12]. Among all GPXs family members, GPX4 is however the only one able to directly reduce cell membrane-oxidized fatty acids and cholesterol. Therefore, this enzyme plays a key role in protecting cells from damage caused by harmful reactive oxygen species (ROS) and is essential for maintaining the integrity of cell membranes.

**Fig. 1:**
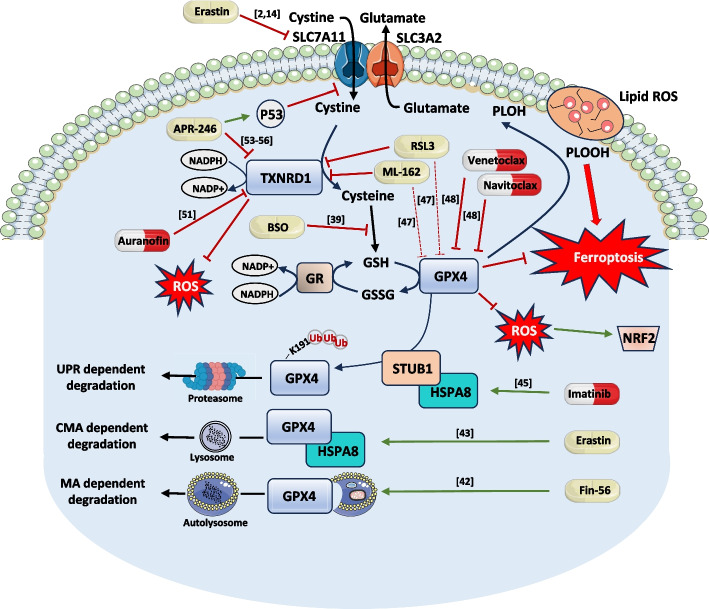
Small molecule inhibitors targeting key detoxifying proteins as molecular probes to target ferroptosis in AML. GPX4 is a key enzyme involved in the inhibition of ferroptosis. Several small molecule inhibitors such as RSL3 and ML-162 have been described as direct inhibitors of this enzyme and as such are able to trigger ferroptosis. More recently, RSL3 and ML-162 were also shown to directly interact with TXNRD1, another important detoxifying enzyme involved in the synthesis of glutathione, an essential cofactor for GPX4 enzymatic activity. In addition, auranofin is also a well characterized inhibitor of TXNRD1 capable of inducing ferroptosis. Venetoclax and Navitoclax are two BH3-mimetics targeting Bcl2 or Blc-2/Bcl-xL respectively. They have been also recently identified as direct inhibitors of GPX4 activity. This is of importance, since these two BH3-mimetics have emerged as indispensable drugs in the armamentarium of AML treatments. Besides inhibiting the cystine/glutamate antiporter (Xc- system), Erastin has been shown to promote the degradation of GPX4 through chaperone-mediated autophagy which also likely contributes to the potent effect of Erastin on ferroptosis. Fin-56, a type III ferroptosis inducer promotes GPX4 degradation through macroautophagy. Interestingly, Imatinib, a tyrosine kinase inhibitor targeting Bcr-Abl and several tyrosine kinase receptors has been reported to increase transcription of STUB1, a GPX4 E3-ligase that triggers the polyubiquitination of lysine 191, thus favoring its degradation by the ubiquitin proteasome system. Finally, APR-246 (PRIMA-1) is a small covalent molecule that can reactivate P53 mutants in AML cell lines. APR-246 was also shown to inhibit the Xc- transport system and more recently to directly target TXNRD1. In line with these findings, APR-246 also induces ferroptosis in AML cell lines and bone marrow cells from AML patients even in the absence of P53. The antileukemic effect of APR-246 is therefore complex and not necessarily linked to P53 expression and activity

The term ferroptosis was introduced in 2012 to describe a novel mode of cell death induced by small molecule inhibitors including Erastin and RSL3 [9]. The hallmarks of ferroptosis include accumulation of membrane lipid hydroxy peroxides, increased intracellular ROS, oxidative stress, and mitochondrial shrinkage. Ferroptosis stand outs among other modes of cell death including apoptosis, autophagic cell death, necrosis and pyroptosis, in that it is impaired by iron chelators such as deferoxamine and ciclopirox and by lipid antioxidants such as ferrostatin-1 and a-tocopherol [13]. Thus, ferroptosis can be defined as a non-apoptotic mode of regulated cell death, driven by iron accumulation and membrane lipid peroxidation when GPX4 activity is inhibited (Fig. 1).

Since then, RSL3 was described as an inhibitor of GPX4 and Erastin was shown to inhibit the Xc- transport system, also called the cystine/glutamate antiporter. The Xc- transport system which is composed of SLC3A2 and SLC7A11 proteins carries cystine into the cytoplasm where it is reduced to cysteine by thioredoxin reductases including thioredoxin reductase 1 (TXNRD1), thus participating in the synthesis of intracellular GSH, an essential cofactor for GPX4 activity. Inhibition of the Xc- transport system by genetic (siRNA, CRISPR) or pharmacological means (Erastin, Sulfasalazine) impairs cystine transport and GSH synthesis, leading to the inactivation of GPX4 and induction of ferroptosis [14].

Ferroptosis is critically dependent on iron metabolism [15]. Ferric ion (Fe^3+^) is taken over by transferrin and is subsequently converted to ferrous ion (Fe^2+^) in the cell [10]. Despite its essential role in life, excessive iron is toxic due to its ability to generate ROS and to trigger cell death. While balanced concentrations of Fe^2+^ are essential for the survival of living organisms, iron overload is responsible for the generation of metabolically toxic ROS and phospholipid hydroxy peroxides through the Fenton reaction, ultimately leading to cell damage and death by ferroptosis [16, 17]. Thus, pharmacological inhibition of cystine/glutamate antiporter, inhibition of GSH levels, inactivation of GPX4 or iron overload are all critical factors leading to cellular ferroptosis. Genetic evidence has highlighted that overexpression or knock out of the *Gpx4* gene resulted in reduction or induction of ferroptosis, respectively [1, 18]. Since then, increasing pieces of evidence have accumulated to imply GPX4 as an important determinant of myeloid cell homeostasis and differentiation and likely of leukemic transformation.

The present review thus focuses on the role of GPX4 in the myeloid lineage, describing how increase in GPX4 expression can impact on myeloid cell homeostasis and transformation and whether GPX4 inhibition could represent an attractive strategy to treat AML patients.

## GPX4 in hematopoietic stem cells (HSCs) and the myeloid lineage

ROS production is one of the major pathways activated following all forms of stress [19]. Stress response proteins thus play a crucial role in HSCs maintenance and recent studies have underpinned the role of antioxidants, such as tempol and N-acetyl cysteine in maintaining the quiescence of HSCs [20]. Moreover, HSCs activation need to be resolved soon after acute stress to protect their integrity and prevent their exhaustion. As mentioned previously, at steady state, HSCs exhibit very low levels of ROS. Once activated, their levels of ROS increase, as messengers to sustain their proliferation and differentiation programs. However, at the end of the stress episode, ROS concentration need to return to normal level to avoid HSCs exhaustion. It was recently shown that antioxidants can prevent loss of HSCs self-renewal potential in several contexts, such as aging or after exposure to low doses of irradiation, suggesting that antioxidants can be used to maintain HSCs functional properties upon culture-induced stress [20, 21].

As a major hydroxy peroxide scavenger, GPX4 can suppress ferroptosis thus playing an essential role in HSCs homeostasis. This was clearly demonstrated using Vav1-GPX4^fl/fl^ mice with a specific deletion of GPX4 in the hematopoietic system. Although GPX4 depletion alone failed to affect HSCs function in vivo*,* Vav1-*Gpx4* knockout mice fed with a vitamin E-depleted diet exhibited a reduced number of HSCs and dysregulated hematopoiesis, mainly due to increased ferroptosis [22]. At odds with the in vivo situation, *Gpx4* depleted mice HSCs accumulated lipid hydroxy peroxides and underwent ferroptosis when cultivated ex vivo. These results demonstrate that GPX4 and Vitamin E can finely tune the lipid redox balance in HSCs, thus preventing them from ferroptosis. In the same vein, it has been reported that ablation of *Gpx4* in HSCs triggers anemia associated with an increase in the fraction of erythroid precursor cells and reticulocytes. Despite increased extramedullary erythropoiesis, reticulocytes failed to mature into red blood cells and accumulated large autophagosomes with engulfed mitochondria [23]. In addition, *Gpx4*-deletion in HSCs led to systemic hepatic iron overload and simultaneous severe iron demand in the erythroid system.

**Fig. 2:**
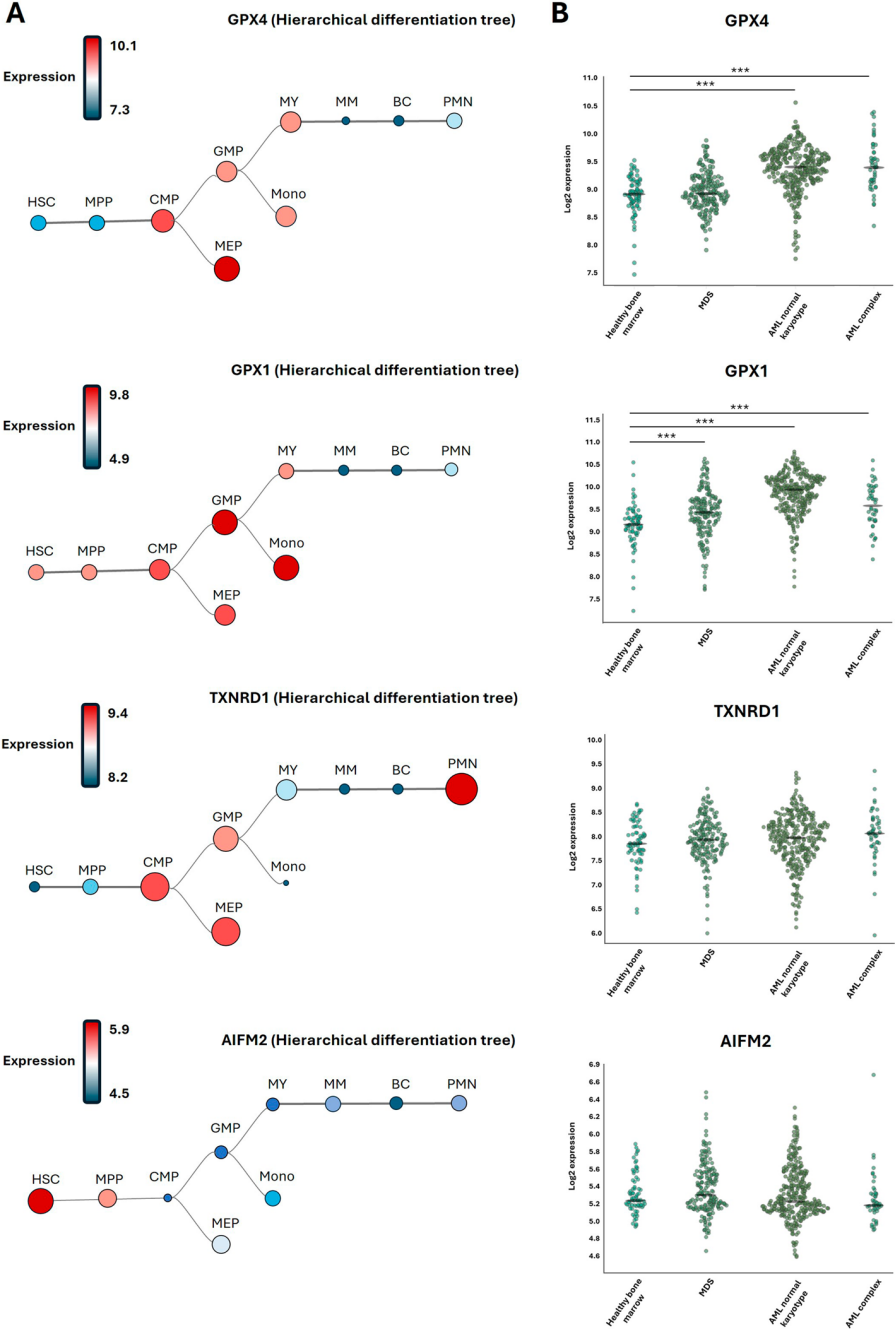
Expression of GPX1, GPX4, TXNRD1 and AIFM2 in the myeloid lineage. **A** GPX1 and GPX4 mRNA expression follow the same trajectories during development of the myeloid lineage. Relatively low in hematopoietic stem cells (HSC) and multipotent myeloid progenitors (MPP), GPX1 and GPX4 mRNA expression increases in common myeloid progenitors (CMP), megakaryocyte erythroid progenitors (MEP), granulocyte myeloid progenitors (GMP), monocytes (Mono) and drastically decreases in polymorphonuclear neutrophiles (PMN). The level of TXNRD1 mRNA expression is comparable to the one of GPX1 and GPX4, at the noticeable exception of Mono and PMN. Indeed, conversely to GPX1 and GPX4, expression of TXNRD1 is lower in monocytes and higher in PMN. AIFM2 mRNA expression is higher in HSCs than in other hematopoietic cells. The data used to generate these Hierarchical Differentiation Tree were obtained from a cohort know as “normal hematopoiesis with AMLs” through the bloodspot website (https://bloodspot.eu/). **B** In a cohort of acute myeloid leukemia (AML) patients GPX4 mRNA expression is increased in patients with both normal and complex karyotypes but unchanged in patients suffering myelodysplastic syndromes (MDS). GPX1 mRNA is elevated in the three subtypes of patients. TXNRD1 and AIFM2 mRNA expression are unaffected in MDS and AML patients. The data used for generating these graphs were obtained from the Microarray Innovations in Leukemia (MILE) study via the bloodspot website (https://bloodspot.eu/)

As mentioned previously, expression of GPX4 and more generally all GPXs proteins are relatively weak in HSCs. During development of the myeloid lineage, GPX1 and GPX4 expression turns out to drastically increase, notably in human common myeloid progenitors (CMP), granulocytes-monocytes progenitors (GMP), megakaryocyte-erythroid progenitors (MEP) and in monocytes, while it remains very low in polymorphonuclear cells (PMN) (Fig. 2). Conversely, GPX2 and 3 expressions are elevated only in the more mature myeloid cells (PMN and monocytes) (Fig. 2). These observations suggest that besides modulating HSCs homeostasis, GPX4 may also have an important role in regulating monocyte and macrophage functions.

The interaction between macrophages and ferroptosis has been the subject of an excellent recent review [24]. It is well established that iron overload and more generally ferroptosis can trigger macrophage polarization, increasing M1-like markers such as IL-6, IL-1, and TNFα, while decreasing M2-like markers [25, 26]. In this line, a direct role for GPX4 in the process of macrophage polarization has been suggested. Indeed, loss of GPX4 expression and activity specifically in monocytes/macrophages triggers ferroptosis in IL-4-driven macrophage differentiation, while sparing inflammatory macrophages, which were able to sustain cell survival and function in the absence of GPX4. This suggests that GPX4-dependent antioxidant defense regulation could be different in various myeloid cell subtypes to finely tune their homeostasis and their differentiation and polarization programs [27, 28]. One may however assume that GPX4 inhibition would promote M1 polarization of macrophages at the expense of M2 polarization. In this context, besides their well described effect on solid tumors, reprogramming tumor resident macrophages using GPX4 inhibitors could be of potential interest in solid cancer and hematopoietic malignancies including AML.

### GPX4 function in myeloid leukemia

Numerous studies have reported the occurrence of a high level of intracellular ROS in different AML cell lines and in bone marrow cells derived from MDS and AML patient’s samples [29–32]. However, keeping an elevated intracellular ROS level within AML cells without altering their viability and proliferation potential requires the establishment of a set of detoxification mechanisms to support their continued growth [33]. In this context, increased expression of detoxifying enzymes may lead to the development of redox adaptative mechanisms that could perturb cell homeostasis and even promote leukemic transformation. In addition, oxidative stress can also trigger DNA damage that leads to genomic instability and modification of the cellular mutational status of AML cells, thus favoring tumor progression [34].

It has been consistently reported that expression of GPX4 is elevated in most of AML cells and cell lines compared to their normal myeloid counterparts [35]. In this line, high expression of GPX4 in AML cells may also favor cellular growth and increase transformation potential. As mentioned previously by keeping ferroptosis under control, GPX4 likely plays an essential role in tuning AML cell homeostasis. Interestingly, high GPX1 and GPX4 expression are correlated to poor prognosis in patients suffering AML, suggesting that targeting one or both proteins could be of high potential therapeutic value [36]. Moreover, in the bio portal TGCA AML cohort, overexpression of both GPX1 and GPX4 which is found in a significant subset of patients is associated with a poor prognosis [36].

Recently Ma et al., using multiparametric analysis of GPX4 and Apoptosis Inducing Factor Mitochondria Associated 2 (AIFM2) also called ferroptosis suppressor protein (FSP1) expression and function identified high expression of both proteins as novel biomarkers of pejorative prognosis in AML [35]. AIFM2 is a NAD(P)H-dependent oxidoreductase that catalyzes reduction of coenzyme Q/ubiquinone-10 to ubiquinol-10, a lipophilic antioxidant that prevents ferroptosis. AIFM2 acts in parallel to GPX4 to suppress phospholipid peroxidation and ferroptosis [37], but independently of cellular glutathione levels. During normal hematopoiesis, AIFM2 expression is low in HSCs and CMP and high in mature myeloid cells such as PMN and monocytes. Of note AIFM2 expression is very high in AML with inversion of chromosome 16 and t (11; q23) translocation, suggesting that targeting of AIFM2 in this peculiar AML subset could be of utmost therapeutic interest.

In a very recent paper, Akiyama et al. investigated ferroptosis in AML cells and identified its mitochondrial regulation as a novel therapeutic vulnerability [38]. As expected, they first showed that GPX4 invalidation induced ferroptosis in AML cells that is associated with mitochondrial lipid peroxidation, culminating in antileukemic effects in vitro and in vivo. They next established that mitochondrial Coenzyme Q counteracted ferroptosis triggered by pharmacological and genetic inhibition of GPX4, suggesting that mitochondrial lipid redox signaling regulates ferroptosis in AML cells. Consistently, Rho0 AML cells, lacking functional electron transport chain (ETC), were found to be highly sensitive to mitochondrial lipid peroxidation and ferroptosis compared to control cells. Furthermore, they established that degradation of ETC proteins through hyperactivation of caseinolytic protease P, synergistically enhanced the anti-AML effects of GPX4 inhibition. Collectively, these data highlight an important role of ETC and mitochondrial lipid redox signaling in GPX4-dependent ferroptosis in AML cells and anticipate that targeting both pathways (i.e. ETC and ferroptosis) could favor eradication of AML cells at least in vitro.

More globally, analysis of GPX4 protein expression levels performed in up to forty different cancers show that besides AML, GPX4 is also elevated in most hematological malignancies including Acute Lymphoblastic Leukemia (ALL), Multiple Myeloma (MM) and Non-Hodgkin's Lymphoma (NHL) [39]. Some solid cancers also exhibited high level of GPX4 expression including melanoma, gastric, endometrial, prostate and colorectal cancers. Most of the time high GPX4 expression correlated with a poor prognosis.

## Targeting GPX4 and ferroptosis in AML

The role of GPX4 in the development of the myeloid lineage and in the initiation and progression of myeloid leukemia remains poorly explored. Given its essential role in the detoxification of lipid hydroxy peroxides, and its overexpression in most of myeloid malignancies, GPX4 inhibition has emerged as a promising therapeutic strategy to induce ferroptosis as a mean of eradicating myeloid leukemia cells. Therefore, the availability and use of compounds capable of interfering with exacerbated detoxification processes and ferroptosis have emerged as major assets in enabling the selective elimination of leukemia cells, while minimizing their impact on healthy ones. Over the last 10 years, major efforts have been made to develop different types of GPX4 inhibitors, and their mechanisms of action have been partially elucidated. Among them RSL3 and ML162 were first described as direct inhibitors of GPX4, while others such as erastin and sulfasazaline were shown to target the Xc- transport system (Fig. 1).

### Inhibition of the Xc- transport system and depletion of cystine

The Xc- transport system, which is composed of SLC7A11 and SLC3A2 proteins imports cystine into the cell while exporting glutamate. Transport Xc plays an essential role in the central nervous system through its ability to regulate glutamate levels [40]. Inside the cell, cystine is reduced to cysteine, a precursor of GSH and an indispensable cofactor for GPX4 activity. Expectedly, depletion of cystine or inhibition of the Xc- antiporter using synthetic small molecule inhibitors including erastin, sulfasalazine, sorafenib and even excess of glutamate triggered cystine depletion, decreased GSH level and reduced GPX4 activity, culminating in ferroptosis induction. Besides inhibiting the Xc- antiporter, erastin is also an activator of the mitochondrial voltage-dependent-anion channel (VDAC) by reversing the inhibitory effect of tubulin on VDAC, thus increasing oxidative phosphorylation, ATP, ROS production and mitochondrial membrane permeabilization. Interestingly, Cunningham et al. reported that inhibition of the Xc- transport system using sulfasalazine, while ineffective as a monotherapy, was highly effective to eliminate AML cell lines when combined with Buthionine sulfoximine (BSO), an inhibitor of glutamyl cysteine synthetase, the first enzyme in the synthesis of GSH. They therefore propose the use of a combination of either sulfasalazine or antioxidant machinery inhibitors along with ROS inducers such as BSO or chemotherapy for further preclinical testing [41]. Importantly, the same authors also reported that AML cells were unable to synthetize cysteine from methionine providing an opportunity to exploit this vulnerability for AML treatment. Of note, AML patients with high SLC7A11 mRNA expression exhibited a drastically lower overall survival than patients with low SLC7A11 expression [42, 43].

### FIN56-induced degradation of GPX4 through chaperone-mediated autophagy

Recently, Sun et al. [44] reported that ferroptosis is a type of autophagy-dependent cell death. Indeed, they showed that Fin56, a type 3 ferroptosis inducer, triggers ferroptosis by promoting GPX4 degradation through autophagy in bladder cell carcinoma. Likewise, Lu et al. showed that the activation of ferroptosis by erastin increased the levels of lysosome-associated membrane protein 2a (LAMP2A) to promote CMA, which, in turn, triggered the degradation of GPX4. Importantly, inhibition of CMA stabilized GPX4 and reduced ferroptosis. Their results suggest that activation of CMA is involved in the execution of ferroptosis through GPX4 degradation in cell lines from different tissue origins [45]. Globally, these findings point to an important role of various forms of autophagy in the regulation of ferroptosis.

### Imatinib-induced GPX4 proteasomal degradation

Imatinib is a tyrosine kinase inhibitor designed to inhibit BCR-ABL activity in chronic myelogenous leukemia (CML) that revolutionized the treatment of this hematopoietic malignancy in the early 2000s [46]. In a recent study Sun et al. showed that imatinib increases lipid ROS and intracellular Fe2+ levels and dampened glutathione production in gastrointestinal tumor cell lines, another imatinib-sensitive tumor, an effect abrogated by ferrostatin-1, an inhibitor of ferroptosis. They also established that overexpression of GPX4 or knock down of STUB1, a GPX4 E3-ligase, alleviated imatinib-mediated ferroptosis induction. Mechanistically, they demonstrated that STUB1 promoted polyubiquitination of GPX4 on lysine 191, leading to its degradation by the ubiquitin proteasome pathway. They finally showed that the combination of RSL3, a ferroptosis inducer with Imatinib synergistically inhibited GIST cell proliferation [47].

### RSL3 and ML162 direct or indirect inhibitors of GPX4 and TXNRD 1?

Although RSL3 and ML162 were first described as direct GPX4 inhibitors [9, 48], several publications recently challenged this notion [49, 50]. Indeed, while efficiently inducing ferroptosis in different tumor cell lines, RSL3 and ML162 were found to also acts on TXRND1, an important detoxifying enzyme whose inhibition can also trigger apoptosis and/or ferroptosis [51, 52]. In addition, the same authors also identified a panel of FDA-approved drugs for their ability to inhibit globally or individually all GPX proteins or only specific ones [50]. Among these inhibitors were BH3 mimetics including Venetoclax and Navitoclax, two molecules widely used for the treatment of MDS and AML. It would be Interesting in future studies to investigate whether Venetoclax and Navitoclax are per se efficient inducers of ferroptosis in AML cell lines and patients. In line with the importance of TXNRD1 in the regulation of ferroptosis and apoptosis in AML cells, auranofin, a TXNRD1 inhibitor, impaired cell viability in AML subtypes dependent on TXRND1 for their protection against ferroptosis [53].

### Concomitant Inhibition of NRF2/GPX4 and NRF2/Bcl2 as new therapeutic strategies in AML

Liu et al., highlighted the important role of NRF2 in AML, where it is critical in detoxifying ROS in combination with GPX4. They showed that concomitant inhibition of NRF2 using ML385 and of GPX4 using FIN56 or RSL3 synergistically eradicate AML through ferroptosis and apoptosis induction, suggesting that such a combination of therapy may represent a promising approach for the treatment of AML. In the same vein, it was recently reported that concomitant Inhibition of NRF2 by ML385 and Bcl2 by venetoclax drastically enhances AML death through ferroptosis [54].

### APR-246 (PRIMA1) a ferroptosis inducer and a TXNRD1 inhibitor?

APR-246 is a small molecule inhibitor which has received lot of attention for its ability to reactivate the transcriptional activity of p53 mutants in different cell types [55]. Further studies have established that APR-246 can also trigger p53-independent cell death in several solid cancers, suggesting that besides p53, other cellular targets might be involved in APR-246 effect [56]. A recent study reported the ability of APR-246 to trigger ferroptosis in different AML cell lines independently of their P53 mutational status [56]. These authors also established that the combination of APR-246 with different compounds and strategies inducing ferroptosis (pharmacological compounds, or genetic inactivation of SLC7A11 or GPX4) had a synergistic effect on the promotion of AML cell death, both in vitro and in vivo. Of note, besides p53 the ability of APR-246 to induce cell death has also been ascribed to the inhibition of TXNRD1 [57, 58]. For instance, the Michael acceptor methylene quinuclidinone, the active form of APR-246, was shown to be a potent and direct inhibitor of both TXNRD1 and Glutaredoxin-1 (GRX1) by reacting with the selenocysteine group in both enzymes. TXNRD1, which catalyzes the reduction of thioredoxin using NADPH as a cofactor, is an important regulator of redox balance in cells [51]. TXNDR1 expression is however relatively homogenous in HSCs and the myeloid lineage and in different AML subtypes. Taken together, these findings suggest that APR-246 could induce tumor cell death through both reactivation of mutant p53 and inhibition of cellular thiol-dependent redox system proteins (GPX4, TXNDR1, GRX1), providing novel combinatorial strategies for AML therapy.

### HA-344 and #231 as dual inhibitors of GPX4 and TXNRD1 in AML cells

In the same line, we recently designed two covalent small molecule inhibitors, that can concomitantly inhibit GPX4 and TXDNR1, two critical proteins involved in the protection of cell from ferroptosis and apoptosis [39]. Both compounds were shown to eradicate AML cell lines through activation of ferroptosis and apoptosis. While GPX4 inhibition by HA-344 or #231 is associated with increased ferroptosis, TXRND1 inhibition disrupts redox homeostasis and promoted increased rate of apoptosis [59, 60]. Importantly, HA-344 and #231 act as specific bio-degraders of GPX4 by a currently unknown mechanism that does not seem to involve the proteasome pathway. Besides AML cell lines these inhibitors also triggered cell death of bone marrow blasts from AML patients without affecting normal blood cells, a property that could be of utmost interest in a therapeutic point of view.

## Conclusions and perspectives

**Fig. 3:**
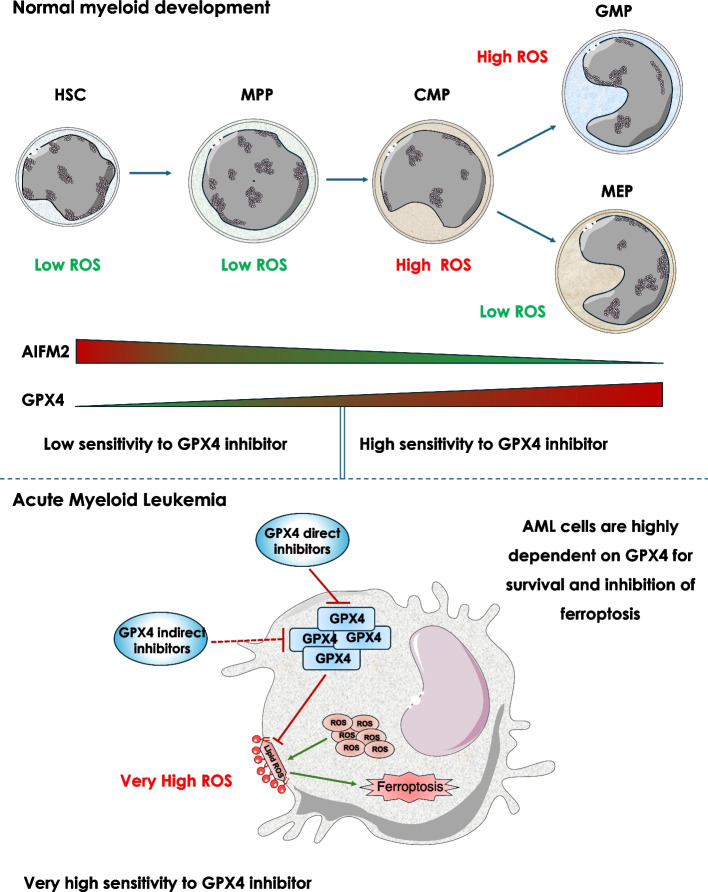
A schematic view of the putative role of GPX4 and AIFM2 in normal hematopoietic cells homeostasis and acute myeloid leukemia. HSCs exhibit relatively low ROS levels, a condition that favors quiescence and self-renewal. Parallelly, HSCs exhibit high level of AIFM2 expression that is therefore more susceptible than GPX4 to protect them from ferroptosis. During engagement of HSCs in the myeloid lineage and more particularly in CMP, GPX4 expression increases drastically, while AIFM2 expression decreased. It has been reported that CMP cells with high ROS levels are more likely to differentiate into GMP, while those with low ROS levels differentiate into MEP [62]. Thus, in CMP, GPX4 could be important to repress ferroptosis, thus favoring differentiation in GMP. The high expression of GPX4 in MEP, is also consistent with a role of GPX4 in MEP protection and with the phenotype of GPX4 deficient mice that exhibit anemia. Finally, the very high expression of GPX4 and GPX1 in all subtypes of AML could participate in the high survival rate and proliferation indexes of these cells and explain their very high sensitivity of GPX4 inhibitor-mediated ferroptosis

Cancer cells use a plethora of strategies to evade therapeutic treatments. Among these, resistance to various types of cell death induced by chemotherapies or targeted therapies is one of the key hallmarks of cancer. Ferroptosis has gained lot of attention, these past few years owing to its importance in a wide number of human pathologies including solid cancers and hematopoietic malignancies. Ferroptosis is a newly described form of cell death induced by membrane lipid peroxidation, whose, therapeutic reinduction in resistant tumor cells could have a major clinical interest. To survive in adverse conditions such as increased ROS production, tumor cells implement a variety of strategies, including increased expression of detoxification enzymes, a condition that can favor their survival and transformation ability. Among the detoxification enzymes whose expression is increased in AML cells, GPX4, GPX1 and TXNRD1 have emerged as novel and promising therapeutic targets. Numerous direct or indirect inhibitors of these enzymes have been developed and mainly characterized in vitro. For convenience, Fig. 1 highlights the complex mechanism of action of such inhibitors in ferroptosis and stress response modulation. In conclusion, GPX4 and AIFM2 may considerably impact myeloid cell development, homeostasis and transformation in AML. In this line, Fig. 3 proposes a schematic view of the putative role of GPX4 and AIFM2 in normal hematopoietic cell homeostasis and acute myeloid leukemia.

Rational targeting of GPX1, GPX4 and TXNRD1 holds high therapeutic potential in different pathological settings, where lipid peroxidation is either harmful or can be triggered for therapeutic purpose, as it is the case in numerous cancers and more particularly AML. However, careful consideration is warranted when employing GPX4 inhibitors and ferroptosis inducers in AML and other cancer types, as the tumor microenvironment significantly influences tumor progression, either by promoting or inhibiting it. Although beneficial in tumor cells, GPX4 inhibition can also trigger ferroptosis-induced death of immune cells in the tumor microenvironment leading to ineffective antitumor response. Moreover, most of the so far documented direct or indirect inhibitors of GPX4 may also affect other cellular processes not directly linked to ferroptosis induction leading to potentially detrimental side effects in patients. The recent discovery of the small molecule N6F11, which binds the tripartite region of Trim25 and promotes proteasome-degradation of GPX4 in pancreatic cancer cells, while sparing immune cells and the anti-tumoral response of the microenvironment, could represent a major clinical breakthrough for the use of GPX4 inhibitor/degrader [61].

## Data Availability

Not applicable.

## Abbreviations

AML: Acute myeloid leukemia
GPX: Glutathione peroxidase
GSH: Glutathione
GSSG: Oxidized glutathione
ROS: Reactive oxygen species
xCT: Glutamate cysteine antiporter
SLC7A11: Solute carrier family 7 member 11
SLC3A2: Solute carrier family 3 member 2
NRF2: Nuclear factor erythroid 2-related factor 2
Fe^2+^: Ferrous ion
Fe^3+^: Ferric ion
HSC: Hematopoietic stem cell
Vav1: Vav guanine nucleotide exchange factor 1
CMP: Common myeloid progenitor
GMP: Granulocyte-monocyte progenitor
MEP: Megakaryocytes-erythroid progenitor
PMN: PolyMorphoNuclear
TGCA: TGCA AML cohort
AIFM2: Apoptosis inducing factor mitochondria associate 2
FSP1: Ferroptosis suppressor protein 1
ETC: Electron transport Chai
VDAC: Voltage-dependent anion channel
BSO: Buthionine sulfoximine
CMA: Chaperone-mediated autophagy
Fin56: Ferroptosis inhibitor 56
CML: Chronic myelogenous leukemia
STUB1: STIP1 Homology And U-Box Containing Protein 1
NFE2L2/NRF2: Nuclear factor (erythroid-derived 2)-like 2
FDA: Food and drug administration
TXNRD1: Thioredoxin reductase 1
GRX1: Glutaredoxin 1
MDS: Myelodysplatic syndromes
CREB: CAMP-response element
SP1: SP1 transcription factor
AP2: Transcription factor AP2
PPARα: Proliferator-activated receptor alpha
KLF2: Krüppel-like transcription factor 2
CREM: CAMP-response element modulator
RSL3: Ras-selective lethal 3
LAMP2: Lysosomal-associated membrane protein 2
ALL: Acute lymphoblastic leukemia
MM: Multiple myeloma
NHL: Non-Hodgkin lymphoma
Il-1: Interleukin 1
Il-6: Interleukin 6
TNFα: Tumor necrosis factor alpha
